# Effects of Single-Walled Carbon Nanotubes on the Development and Reproductive Performance of *Tetranychus turkestani*

**DOI:** 10.3390/insects17030284

**Published:** 2026-03-05

**Authors:** Qiancheng Wei, Xiaojun Wang, Kedi Zhao, Heli Qu, Chunjuan Wang, Feng Liu, Yiying Zhao

**Affiliations:** College of Agriculture, Shihezi University, Shihezi 832000, China; 20232112078@stu.shzu.edu.cn (Q.W.); thehousejustwxj@163.com (X.W.); zhaokedibs@163.com (K.Z.); quheli2@163.com (H.Q.); wchunjuan@126.com (C.W.)

**Keywords:** *Tetranychus turkestani*, age-stage, two-sex life table analysis, single-walled carbon nanotubes, pest management, nanotoxicity

## Abstract

Carbon nanotubes (CNTs) show strong potential for improving crop productivity and stress resilience, but their direct effects on agricultural pests require careful evaluation. Here, we assessed single-walled CNTs (SWCNTs) on the spider mite *Tetranychus turkestani* (Acari: Tetranychidae) using an age-stage, two-sex life table. Leaf disks treated with SWCNTs (0.004, 0.04, 0.2, 0.4 mg/mL) were used to measure development, survival, and reproduction. SWCNT exposure significantly prolonged pre-adult development and reduced female fecundity in a concentration-dependent manner. Population parameters (*r*, *λ*) declined significantly in treated groups, while mean generation time (*T*) increased with concentration. At 0.4 mg/mL, fecundity dropped to 40.57 ± 2.42 offspring per female and population projection was lowest. Overall, SWCNTs inhibited mite development and reproduction in a concentration-dependent manner, providing a demographic basis for ecological risk assessment and suggesting potential utility as a tool or carrier system for integrated spider mite management.

## Simple Summary

Spider mites are notorious pests that rapidly develop resistance and are difficult to control in many crops. Concurrently, carbon-based nanomaterials like single-walled carbon nanotubes (SWCNTs) are emerging as promising tools in agriculture. However, their direct effects on pest population growth remain poorly understood. In this study, we investigated how SWCNTs affect the development and reproduction of the spider mite *Tetranychus turkestani*. Using a detailed life table approach, we found that exposure to SWCNTs prolonged the juvenile development time and reduced the egg-laying capacity of the mites. Key indicators of population growth were also suppressed. These results provide crucial evidence that SWCNTs can inhibit spider mite population growth. This study not only contributes to the environmental risk assessment of nanomaterials but also provides a demographic basis for evaluating their potential utility as innovative components in nano-enabled pest management strategies.

## 1. Introduction

Nanomaterials (NMs) are defined as materials with at least one dimension smaller than 100 nm in three-dimensional space, and they exhibit distinctive physicochemical characteristics such as small particle size, large specific surface area, and high reactivity [1,2]. In recent years, nanotechnology has created new opportunities to improve agricultural productivity through applications such as nanocapsules, highly efficient insecticides, and fertilizers. It has also been considered an environmentally friendly alternative to conventional pesticides for pest control [3]. Among various nanomaterials, carbon-based nanomaterials (CNMs) play a pivotal role in agricultural production [4,5]. In particular, carbon nanotubes (CNTs) have attracted substantial attention due to their unique rigidity, strength, and elasticity, as well as superior performance compared with other fibrous materials [6]. Increasing evidence indicates that CNMs—especially CNTs—can readily penetrate plant cell membranes and alter plant physiological and morphological traits, thereby showing pronounced potential in promoting crop growth, enhancing stress tolerance, and serving as carriers for fertilizers and pesticides to improve their utilization efficiency [7]. For instance, CNMs can function as smart delivery systems to transport DNA molecules or oligonucleotides into plant cells, enabling efficient gene transfer [8]. In addition, modified CNTs or graphene oxide (GO)-based sensors have been widely applied to detect pesticide residues in the environment, further highlighting the value of CNMs for sustainable agriculture [7,8].

Among carbon-based nanomaterials, single-walled carbon nanotubes (SWCNTs) are particularly appealing because of their high aspect ratio, small size (diameter ~0.8–1.2 nm), and unique cylindrical nanostructure [9,10]. Compared with multi-walled carbon nanotubes (MWCNTs), the minimum dimension of SWCNTs is below the size-exclusion limit of the plant cell wall (approximately 20 nm), allowing SWCNTs to passively traverse plant cell membranes in a species-independent and non-integrative manner and thereby achieve efficient biomolecule delivery [8]. Beyond their role as delivery systems, the direct application of SWCNTs onto plants has been shown to elicit a range of physiological responses. Studies have reported both positive effects, such as enhanced seed germination, growth promotion, and increased photosynthetic activity in various plant species, as well as negative effects, including phytotoxicity, oxidative stress, and growth inhibition at higher concentrations. These dual effects are often concentration-dependent and vary with plant species and SWCNT properties. Given that *T. turkestani* feeds on leaf mesophyll cells, any SWCNT-induced changes in plant physiology or leaf surface properties could indirectly influence mite performance, a factor that should be considered when interpreting the direct toxicity of SWCNTs on the pest. Accumulating studies have shown that SWCNTs can serve as intelligent delivery platforms for DNA, oligonucleotides, or siRNA, markedly improving the application efficiency of agrochemicals and nucleic acids [8,11]. For example, SWCNT-mediated siRNA delivery can achieve up to 95% gene silencing efficiency in plant leaves, and the near-infrared fluorescence of SWCNTs facilitates tracking of nanoparticle–cargo complexes deep within plant tissues [7]. These advantages suggest that SWCNTs may provide higher specificity, stability, and environmental compatibility for pest and disease management, and they may be more promising than conventional MWCNTs or other nanocarriers for controlled-release pesticide delivery and RNA interference (RNAi) platforms [2,12].

The cotton spider mite, *Tetranychus turkestani* (Acari: Tetranychidae), is among the most destructive piercing–sucking pests worldwide, with a host range exceeding 1100 plant species [13]. These mites feed on the contents of mesophyll cells and damage leaf tissues through needle-like mouthparts, causing cell death and chlorotic spots and ultimately resulting in substantial losses in crop yield and quality. Under optimal conditions (e.g., 25–30 °C and 45–55% relative humidity), the developmental cycle of *T. turkestani* can be as short as 7 days, potentially allowing numerous generations per year. Its fecundity is also notably high. This short life cycle and high reproductive capacity accelerate the evolution of pesticide resistance, making spider mites among the most pesticide-resistant arthropods [13,14]. Consequently, conventional insecticides/acaricides such as organophosphates and pyrethroids often show limited efficacy and may lead to residue accumulation and ecological toxicity [3,15]. Therefore, there is an urgent need to develop novel and sustainable control strategies [16].

Despite the promising prospects of carbon nanomaterials in agriculture, systematic assessments of their toxicological effects in agroecosystems—particularly their biological impacts on pest species—remain insufficient [17]. Existing studies have largely focused on a small number of model insects or beneficial insects, and the outcomes are inconsistent [18,19]. Notably, comprehensive investigations on the effects of CNTs on major agricultural pests, especially piercing–sucking pests such as spider mites, with respect to development, reproduction, and population dynamics are still scarce [20]. Life table analysis is widely recognized as a robust approach for evaluating the long-term demographic consequences of external stressors, as it provides an integrative assessment of development, survival, fecundity, and population growth parameters [21,22]. Accordingly, the present study employed the age–stage, two-sex life table method to systematically examine, for the first time, the potential effects of non-functionalized SWCNTs at different concentrations on the development and reproductive performance of *Tetranychus turkestani* using age-stage, two-sex life table analysis. This work not only provides experimental evidence for elucidating the biological effects of SWCNTs but also offers theoretical support for their potential application as pesticidal agents and dsRNA carriers for effective spider mite management, thereby promoting the safe and rational use of nanomaterials in green plant protection.

## 2. Materials and Methods

### 2.1. Rearing of the Test Spider Mite Population

A laboratory colony of *Tetranychus turkestani* was established from individuals collected in 2022 from the experimental fields of the College of Agriculture, Shihezi University (China). Species identification was performed based on morphological characteristics of adult females and males using a taxonomic key for Tetranychidae [16]. To ensure purity, the identity of the colony was periodically confirmed by examining key morphological features (e.g., the shape of the aedeagus in males) under a phase-contrast microscope. Voucher specimens have been deposited in the insect collection of the College of Agriculture, Shihezi University. The colony was maintained in a controlled-environment chamber at the Insect Physiology Laboratory, Shihezi University, using potted common bean (*Phaseolus vulgaris*) plants as the host. Rearing conditions were 25 ± 1 °C, 60 ± 5% relative humidity, and a 16:8 h light/dark photoperiod. To ensure continuous food availability and maintain colony performance, fresh mite-free bean seedlings were introduced every two weeks. No pesticides or other chemical treatments were applied during colony maintenance. For bioassays, adult females were randomly selected from the colony and used only if they were vigorous, undamaged, and visually uniform in size.

### 2.2. Materials and Reagents

Single-walled carbon nanotubes (SWCNTs) used in this study were purchased from Jiangsu Xianfeng Nano Material Technology Co., Ltd. (Nanjing, China). The SWCNTs (product no. XFS21) had a purity of >95%, a diameter of 1–2 nm, a length of 1–3 μm, and an electrical conductivity of >100 S/cm.

### 2.3. SWCNTs, Preparation, and Characterization

SWCNT suspensions were prepared at 0.004, 0.04, 0.2, and 0.4 mg/mL. For each concentration, an accurately weighed amount of SWCNT powder was added to 50 mL of deionized water in a glass beaker, followed by dispersion using an ultrasonic cleaner (KQ-500DE, Kunshan Ultrasonic Instruments Co., Ltd., Kunshan, China) at 40 kHz and 500 W. To minimize heat buildup, sonication was performed in an intermittent mode (5 min on, 2 min off) for a total of 30 min. After sonication, dispersion quality was visually assessed to confirm a homogeneous suspension without obvious aggregation or sedimentation. All suspensions were used within 24 h of preparation or stored in sealed glass bottles at room temperature until use.

The morphology of SWCNTs was characterized by transmission electron microscopy (TEM; Hitachi HT7800, Hitachi High-Tech Corporation, Tokyo, Japan) and scanning electron microscopy (SEM; Hitachi Regulus 8100, Hitachi High-Tech Corporation, Tokyo, Japan). For TEM, diluted SWCNT suspensions were drop-cast onto carbon-coated copper grids and air-dried at room temperature. For SEM, samples were mounted on aluminum stubs and sputter-coated with gold prior to imaging. The surface charge (zeta potential) of SWCNTs in aqueous suspension (0.02 mg/mL) was measured using a Zetasizer Nano ZS instrument (Malvern Panalytical Ltd., Malvern, UK). Functional groups on the SWCNT surface were analyzed by Fourier-transform infrared spectroscopy over the range of 4000–400 cm^−1^.

### 2.4. Host Plants and Treatment Application

Common bean plants were grown from seed in potting substrate under the same controlled conditions used for mite rearing. Plants bearing three to four fully expanded true leaves were selected for experiments.

Bioassays were conducted using a leaf-disk dipping method. Leaf disks (28 mm diameter) were excised from healthy, mite-free bean leaves with a stainless-steel cork borer. Disks were individually immersed for 10 min in SWCNT suspensions at the four test concentrations or in deionized water (control). After dipping, disks were removed carefully and placed abaxial side up on sterile filter paper, then air-dried at room temperature until no visible droplets remained (approximately 2 h). Each treated or control disk was transferred to a transparent plastic Petri dish lined with a moistened sponge to maintain high humidity. A damp cotton thread was gently placed around the disk margin to prevent mite escape.

### 2.5. Life Table Study and Data Collection

Sublethal effects of SWCNTs were evaluated using the age–stage, two-sex life table approach. Approximately 100 healthy adult females were randomly collected from the stock colony and transferred onto fresh, untreated bean leaves for oviposition. After 12 h, the females were removed, and newly laid eggs (0–12 h old) were used as the experimental cohort.

For each treatment (four SWCNT concentrations plus the control), 70 eggs were randomly selected. To prevent mites from escaping and to allow individual tracking, each egg was reared in an independent arena. Specifically, 70 individual leaf disks (28 mm diameter) were prepared for each treatment, with each disk placed into a separate 3.5 cm diameter plastic Petri dish lined with moistened filter paper to maintain humidity. A thin layer of water-saturated cotton was wrapped around the edge of each leaf disk to act as a barrier, creating a circular arena on each disk. Thus, each of the 70 eggs per treatment was reared on its own individual leaf disk within its own Petri dish, allowing for precise daily observations of each mite’s development and reproduction throughout its lifetime. Petri dishes were maintained in an environmental chamber at 25 ± 1 °C, 60 ± 5% relative humidity, and a 16:8 h light/dark photoperiod. To ensure consistent exposure to fresh SWCNT residues and maintain leaf quality, the treated or control leaf disks were replaced every 2 days. During replacement, the old leaf disk was carefully removed from the Petri dish. Using a fine camel-hair brush, the mite (whether egg, larva, nymph, or adult) was gently transferred onto the center of a newly prepared, correspondingly treated or control leaf disk. The new disk was then placed into the same Petri dish setup. This process ensured minimal disturbance and continuous monitoring of each individual. Individuals were examined daily at a fixed time, and developmental stage (egg, larva, protonymph, deutonymph, and adult) was recorded; molting was confirmed by the presence of exuviae. Survival was checked daily. For each adult female, daily fecundity was recorded and eggs were removed promptly until death. Adult male longevity was recorded in the same manner.

### 2.6. Life Table Analysis

The raw data on individual development, survival, and daily fecundity were analyzed based on the age-stage, two-sex life table theory using the TWOSEX-MSChart software (version 2.0) computer program [20]. This approach accounts for both age- and stage-specific differences among individuals, which is critical for arthropods like mites where developmental rates vary.

From these data, we calculated the following parameters: age-stage-specific survival rate (*s_xj_*), age-specific survival rate (*l_x_*), age-stage-specific fecundity (*f_xj_*), age-specific fecundity (*m_x_*), and age-specific maternity (*l_x_m_x_*). The population parameters were calculated as follows: net reproductive rate (*R*_0_) representing the total number of female offspring produced per female over its lifetime; intrinsic rate of increase (*r*) estimated using the Euler-Lotka equation; finite rate of increase (*λ* = er); and mean generation time (*T* = ln*R*_0_/*r*).

The standard errors of all developmental times, longevity, fecundity, and population parameters were estimated using the bootstrap procedure with 100,000 resamplings to ensure robust statistical inference [21]. Differences among treatments were compared using the paired bootstrap test at the 5% significance level, implemented in the TWOSEX-MSChart software.

### 2.7. Population Projection

The projected population growth of *Tetranychus turkestani* at various concentrations (0.004, 0.04, 0.2, and 0.4 mg/mL) and in the control group was conducted using the methodology provided by Chi [21,23]. The estimation of future population size and age-stage structure was accomplished through the application of the TIMING-MSChart computer program [24].

## 3. Results

Characterization of SWCNTs. The morphology, surface charge, and functional groups of the SWCNTs were analyzed. The results confirmed the tubular structure and negative surface charge (zeta potential: −12.9 ± 0.43 mV) of the SWCNTs, indicative of good dispersion stability. Detailed characterization data, including SEM/TEM images, zeta potential distribution, and FTIR spectrum, are provided in Figure S1 (Supplementary Materials).

### 3.1. Sublethal Effects of SWCNTs on Developmental and Reproductive Traits

**Figure 1:**
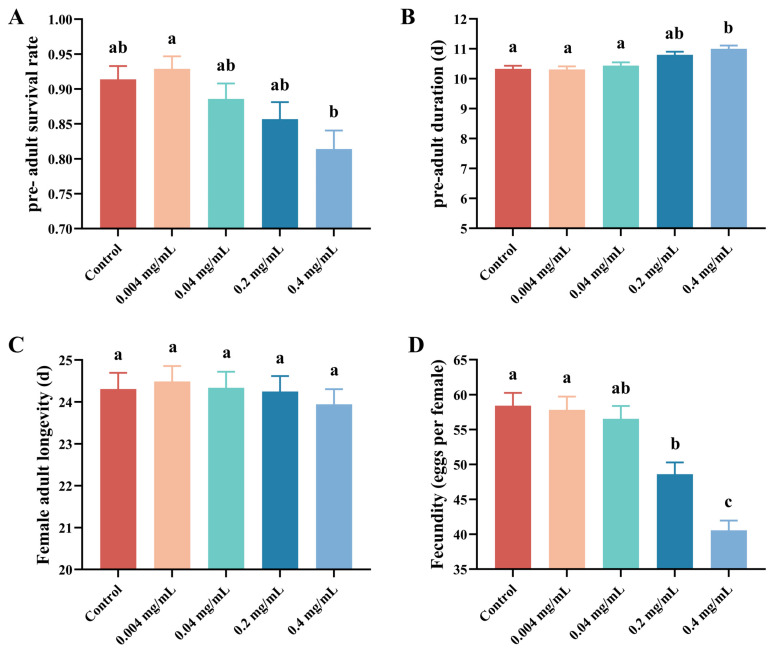
Effects of different concentrations of single-walled carbon nanotubes (SWCNTs) on the demographic parameters of *Tetranychus turkestani*. (**A**) Pre-adult survival rate. (**B**) Pre-adult duration. (**C**) Female adult longevity. (**D**) Total fecundity. Values are presented as mean ± standard error (SE). Different lowercase letters above bars indicate significant differences among treatments based on a paired bootstrap test with 100,000 replications (*p* < 0.05).

The effects of SWCNTs on life-history traits of *Tetranychus turkestani* exhibited a clear concentration-threshold pattern (Figure 1). Overall, at concentrations ≤ 0.04 mg/mL (0.004 and 0.04 mg/mL), SWCNT exposure did not significantly alter immature developmental duration, female adult longevity, or reproductive output relative to the control, indicating a concentration range where minimal biological disruption was observed under our experimental conditions. For example, total fecundity at 0.04 mg/mL was 56.54 ± 3.19 eggs per female, which was not statistically different from the control (58.43 ± 3.17 eggs per female) (Figure 1).

When the SWCNT concentration increased to ≥0.2 mg/mL, pronounced biological suppression became evident, suggesting a transition from a “carrier-compatible” range to a concentration domain that imposes substantial interference on the mite. Specifically, immature development was significantly prolonged, with the pre-adult duration reaching 11.00 ± 0.19 days at 0.4 mg/mL compared with 10.33 ± 0.17 days in the control (Figure 1). Reproductive performance was more strongly inhibited: fecundity decreased to 48.63 ± 2.90 and 40.57 ± 2.42 eggs per female at 0.2 and 0.4 mg/mL, respectively, both statistically significantly lower than the control (paired bootstrap test, *p* < 0.05) (Figure 1D). From a biological perspective, this represents a 16.8% and 30.6% reduction in reproductive output at the two highest concentrations, respectively, indicating a substantial impact on individual fitness. In contrast, female adult longevity remained largely unchanged (23.94 ± 0.63 days at 0.4 mg/mL vs. 24.31 ± 0.67 days in the control; not significant) (Figure 1). Pre-adult survival showed a concentration-dependent decline, reaching the lowest value at 0.4 mg/mL (0.81 ± 0.05), indicating that elevated concentrations impose a stronger penalty on early developmental stages (Figure 1A).

### 3.2. Effects of SWCNTs on Population Parameters

**Figure 2:**
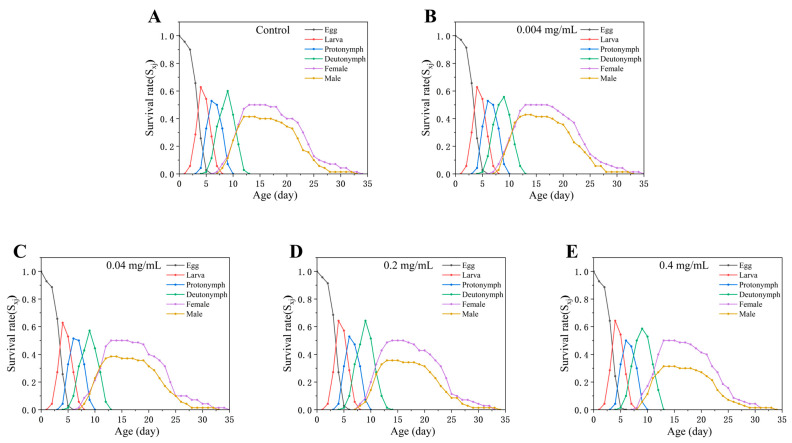
Age-stage-specific survival rate (*s_xj_*) of *Tetranychus turkestani* exposed to different concentrations of single-walled carbon nanotubes (SWCNTs). *s_xj_* shows the probability that a newly laid egg will survive to age *x* and stage *j*. The curves for different stages (egg, larva, nymph, and adult) within each treatment are distinguished by colors as shown in the legend. (**A**) Control; (**B**) 0.004 mg/mL; (**C**) 0.04 mg/mL; (**D**) 0.2 mg/mL; (**E**) 0.4 mg/mL.

Due to inter-individual variation in developmental rates, survival trajectories of *Tetranychus turkestani* typically exhibit pronounced stage overlap. The age–stage-specific survival rate (*s_xj_*) shown in Figure 2 describes the probability that a newborn individual survives to age *x* and stage *j*. The substantial overlap among stage-specific curves highlights the necessity of evaluating stage-specific survival rather than simplifying the life cycle into non-overlapping stages based on mean durations. With respect to treatment effects, *s_xj_* curves under the lower concentrations (0.004 and 0.04 mg/mL) were broadly comparable to those of the control, whereas a general downward shift in *s_xj_* became evident at concentrations ≥ 0.2 mg/mL, indicating that higher SWCNT levels impose stronger survival pressure across developmental stages (Figure 2).

**Figure 3:**
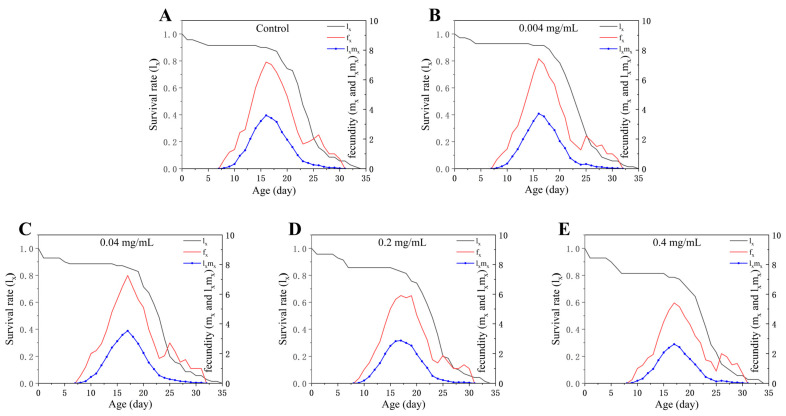
Age-specific survival rate (*l_x_*), age-specific fecundity (*f_x_*), and age-specific maternity (*l_x_m_x_*) of *Tetranychus turkestani* exposed to different concentrations of single-walled carbon nanotubes (SWCNTs). *l_x_* is the probability that a newly laid egg survives to age *x*; *f_x_* is the mean number of eggs produced per individual at age *x*; *l_x_m_x_* represents the age-specific maternity (i.e., expected offspring production weighted by survivorship) at age *x*. (**A**) Control; (**B**) 0.004 mg/mL; (**C**) 0.04 mg/mL; (**D**) 0.2 mg/mL; (**E**) 0.4 mg/mL.

Age-specific survival (*l_x_*), fecundity (*f_x_*), and age-specific maternity (*l_x_m_x_*) under different treatments are presented in Figure 3. In the control, *f_x_* and *l_x_m_x_* on day 16 were 7.2 and 3.6, respectively; by contrast, at 0.4 mg/mL these values decreased to 4.91 and 2.39 on the same day (Figure 3), accompanied by a reduction in *l_x_* from 0.90 (control) to 0.79 (0.4 mg/mL) (Figure 3). These results indicate that at higher SWCNT concentrations, reproductive output and survival maintenance decline concurrently at a critical reproductive time point, which may translate into adverse consequences for population dynamics.

**Figure 4:**
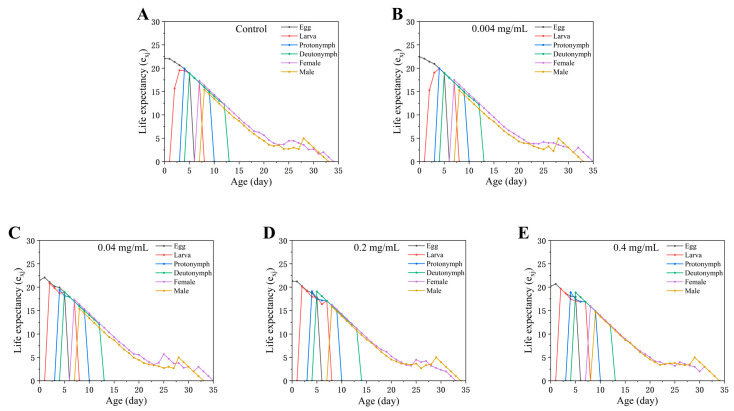
Age-stage-specific life expectancy (*e_xj_*) of *Tetranychus turkestani* exposed to different concentrations of single-walled carbon nanotubes (SWCNTs). *e_xj_* represents the expected remaining lifespan (in days) for an individual that has already survived to age *x* and stage *j*. (**A**) Control; (**B**) 0.004 mg/mL; (**C**) 0.04 mg/mL; (**D**) 0.2 mg/mL; (**E**) 0.4 mg/mL.

The age–stage-specific life expectancy (*e_xj_*) (Figure 4) represents the expected remaining lifespan of an individual at age *x* and stage *j*. Overall, *e_xj_* decreased with increasing SWCNT concentration: adult life expectancy was highest in the control (17.31 days) and lowest at 0.4 mg/mL (15.94 days) (Figure 4). For example, a 10-day-old female adult had an expected remaining lifespan of 8.31 days in the control, compared with 7.18 days at 0.4 mg/mL (Figure 4). Because life expectancy is calculated directly from *s_xj_* and does not require the population to reach a stable age–stage distribution, it provides a sensitive measure of treatment-specific differences in survival prospects.

**Figure 5:**
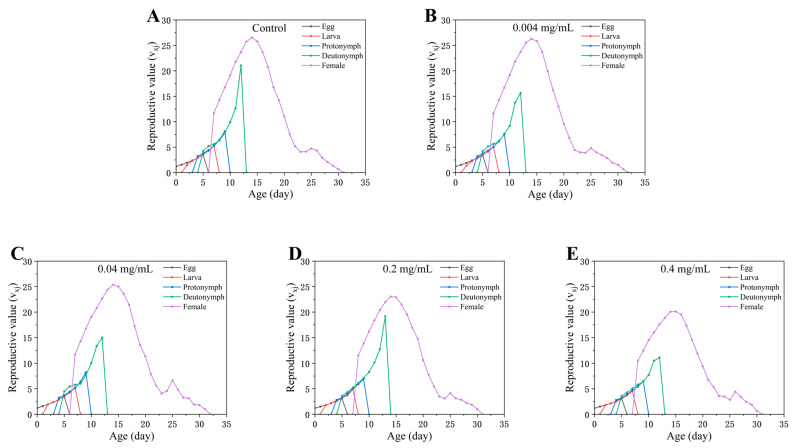
Age-stage-specific reproductive value (*v_xj_*) of *Tetranychus turkestani* exposed to different concentrations of single-walled carbon nanotubes (SWCNTs). *v_xj_* reflects the contribution of an individual at age *x* and stage *j* to the future population growth. The unit is day^−1^. (**A**) Control; (**B**) 0.004 mg/mL; (**C**) 0.04 mg/mL; (**D**) 0.2 mg/mL; (**E**) 0.4 mg/mL.

The age–stage-specific reproductive value (*v_xj_*) quantifies the contribution of individuals at a given age *x* and stage *j* to future population growth (Figure 5). The peak *v_xj_* for female adults on day 8 reached 26.55 in the control, but declined to 20.11 at 0.4 mg/mL (Figure 5). Moreover, *v_xj_* values across stages showed an overall decreasing trend with increasing SWCNT concentration, indicating that high-concentration exposure reduces the contribution of females during the key reproductive window to subsequent population increase (Figure 5).

### 3.3. Effects of SWCNTs on Life Table Parameters

**Figure 6:**
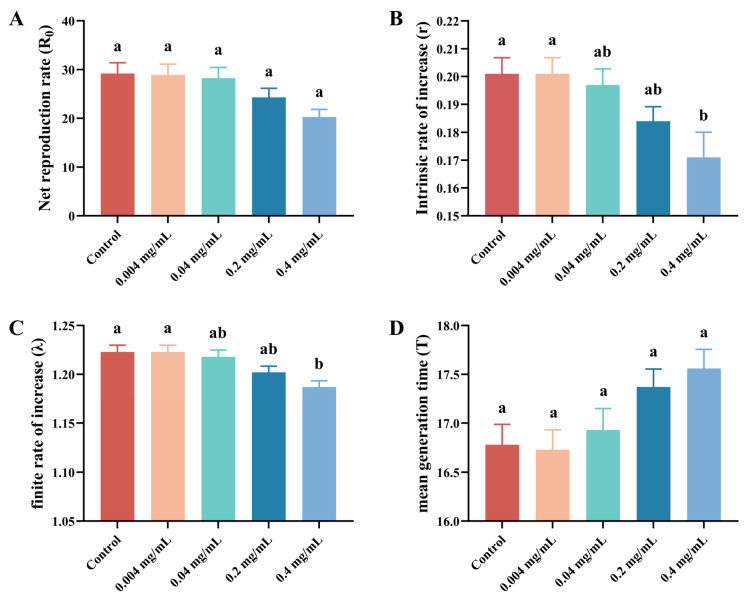
Effects of different concentrations of single-walled carbon nanotubes (SWCNTs) on the population growth parameters of *Tetranychus turkestani*. (**A**) Net reproductive rate (*R*_0_, offspring per individual). (**B**) Intrinsic rate of increase (*r*, day^−1^). (**C**) Finite rate of increase (*λ*, day^−1^). (**D**) Mean generation time (*T*, days). Values are presented as mean ± SE. Different lowercase letters above bars within each subfigure indicate significant differences among treatments (paired bootstrap test, *p* < 0.05).

**Figure 7:**
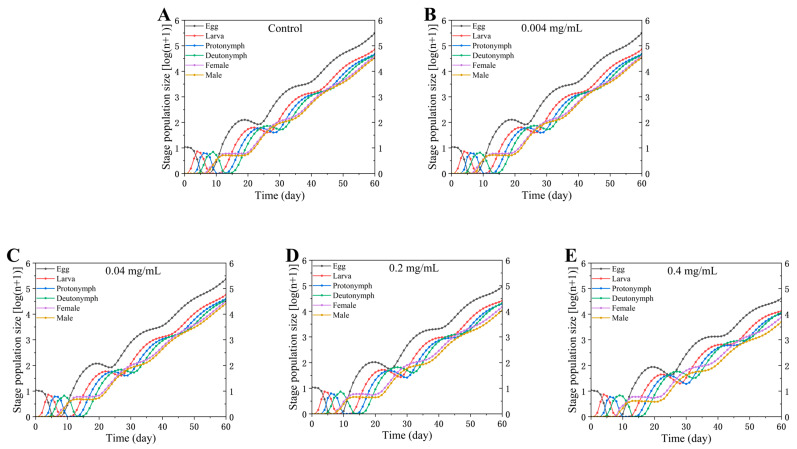
Population projection of *Tetranychus turkestani* over 60 days based on the age-stage, two-sex life table data under different concentrations of single-walled carbon nanotubes (SWCNTs). The curves project the total number of individuals in the population over time. (**A**) Control; (**B**) 0.004 mg/mL; (**C**) 0.04 mg/mL; (**D**) 0.2 mg/mL; (**E**) 0.4 mg/mL.

At the population-parameter level, the effects of SWCNTs on population parameters also exhibited a clear concentration-threshold pattern (Figure 6). The net reproductive rate (*R*_0_) was highest in the control (29.21 ± 3.82) and showed an overall declining trend with increasing SWCNT concentration, suggesting a cumulative inhibitory effect on lifetime reproductive success (Figure 6). In parallel, the intrinsic rate of increase (*r*) and the finite rate of increase (*λ*)—integrative indices reflecting the inherent population growth potential—were comparable to the control under low concentrations (0.004 and 0.04 mg/mL), whereas declines became more pronounced at higher concentrations; a statistically significant reduction was observed only at the highest concentration (0.4 mg/mL) (paired bootstrap test, *p* < 0.05) (Control: *r* = 0.2011 ± 0.0097, λ = 1.2228 ± 0.0119; 0.4 mg/mL: *r* = 0.1714 ± 0.0090, λ = 1.1870 ± 0.0106) (Figure 6). This corresponds to a 14.8% reduction in the intrinsic rate of increase, which, in demographic terms, would substantially slow population growth over multiple generations, as illustrated in the population projection (Figure 7). These results indicate that when SWCNT concentrations remain below the high-concentration threshold (e.g., ≤0.2 mg/mL), the population can largely maintain a growth potential comparable to the control, although reproduction-related traits already show a downward tendency. In contrast, at 0.4 mg/mL, population growth capacity is significantly suppressed.

Mean generation time (*T*) displayed an opposite pattern, increasing with concentration and reaching its maximum at 0.4 mg/mL (17.56 ± 0.34) relative to the control (16.78 ± 0.36) (Figure 6), consistent with the developmental delay induced by high-concentration exposure. Concordantly, fecundity declined monotonically with increasing concentration: the control exhibited the highest egg production (58.43 ± 3.17), whereas the 0.4 mg/mL treatment showed the lowest (40.57 ± 2.42) (Figure 6). Regarding development and survival metrics, the 0.4 mg/mL treatment prolonged the pre-adult developmental period (11.00 ± 0.19), and both pre-adult survival and female adult longevity showed declining trends (Figure 6), collectively indicating that high-concentration SWCNT exposure compromises individual fitness and reduces population growth potential (Figure 6).

### 3.4. Population Projection Based on Life Table Data

To visualize the demographic consequences of SWCNT exposure over an extended time scale, population growth and the age–stage structure of *Tetranychus turkestani* over a 60-day period were simulated using the TIMING-MSChart program (Figure 7). The control group exhibited the fastest population increase and the largest projected final population size. As SWCNT concentration increased, population growth progressively slowed and the final population size declined accordingly, indicating a clear concentration-dependent inhibitory effect. Notably, population growth was most strongly suppressed at 0.4 mg/mL, where the projected number of adult females was the lowest (89,094) (Figure 7), consistent with the reduced population growth parameters (e.g., *r* and *R*_0_) observed under this treatment.

## 4. Discussion

Nanomaterials, functioning either as pesticide synergists or as novel active ingredients, offer new technological avenues for sustainable pest management by improving cargo stability, bioavailability, and target accessibility [2,3]. Against this backdrop, clarifying the intrinsic effects of nanomaterials on pests and mites is essential for defining safe-use boundaries and refining their functional positioning [25,26]. Here, using age–stage, two-sex life table analysis combined with population projection, we systematically evaluated the effects of single-walled carbon nanotubes (SWCNTs) on the development, reproduction, and population growth of *Tetranychus turkestani*. Our control group exhibited a fecundity of 58.43 ± 3.17 eggs per female and an intrinsic rate of increase (*r*) of 0.2011, indicating a healthy and highly reproductive population. These values are within the range of variability reported for *T. turkestani* but somewhat lower than the fecundity observed by Bazazzadeh et al. [14]. This discrepancy could be attributed to differences in host plant species (common bean in our study vs. other hosts in theirs), experimental conditions, or mite strain, highlighting the plasticity of *T. turkestani* life-history traits. Nonetheless, the robust population growth parameters in our control provide a reliable baseline for assessing the sublethal effects of SWCNTs. Our results demonstrate a pronounced concentration-dependent response characterized by a clear boundary between a “low-concentration relatively safe window” and “high-concentration marked suppression.” Specifically, low concentrations (≤0.04 mg/mL) did not significantly affect key population growth parameters (*r* and *λ*); fecundity became significantly suppressed once concentrations reached ≥0.2 mg/mL; and only at the highest concentration (0.4 mg/mL) did *r* and *λ* decline significantly, accompanied by the strongest inhibition in population projection trajectories. The identification of this statistically defined “no-observed-significant-effect concentration window” under laboratory conditions represents a central finding of the present study. From a statistical perspective, this range can be considered a “no-observed-significant-effect concentration window” under our laboratory conditions. Ecologically, this finding suggests that if SWCNTs were to be used as a component in pest management strategies (e.g., as a potential carrier for other active ingredients), concentrations at or below this threshold would minimize direct impacts on mite population growth, thereby allowing clearer attribution of effects to the co-delivered agent. However, it is important to emphasize that this statistical threshold is derived from controlled laboratory conditions and its translation to field applications would require validation under more complex environmental scenarios.

From the perspective of material properties and effective exposure, SWCNTs in this study exhibited a distinctly negative surface charge (zeta potential: −12.9 ± 0.43 mV). SEM/TEM observations indicated a tubular/rod-like morphology with generally good dispersion, and FTIR spectra supported the presence of relevant surface functional group peaks. Negative surface charge typically enhances dispersion stability by promoting electrostatic repulsion among particles; however, partial aggregation may still occur under specific ionic strengths or organic-matter backgrounds, altering effective exposure dose and bioavailability and thereby influencing the apparent magnitude and consistency of ecotoxicological effects [15,27]. Therefore, under foliar exposure scenarios, dispersion–aggregation dynamics may represent an important contextual factor underlying the differentiation of effect strength across concentrations, especially near threshold ranges [15,28].

At the individual level, the observed developmental delay and reproductive suppression are consistent with phenotypic effects reported in other studies of nanomaterial-exposed arthropods [15,20,29]. While our study did not directly measure physiological or molecular mechanisms, based on the existing literature, potential explanations for these effects include oxidative stress induction, physical disruption of digestive tissues, or interference with nutrient assimilation following ingestion of SWCNTs with plant sap [15,29,30]. Further studies employing targeted biochemical assays or transcriptomic analyses would be needed to confirm these mechanistic hypotheses in *T. turkestani*. Such stress burdens may shift energy investment away from growth and reproduction toward maintenance and repair, manifesting phenotypically as prolonged pre-adult development and reduced fecundity. In line with this interpretation, reproductive output decreased persistently once concentrations reached ≥0.2 mg/mL and was most pronounced at 0.4 mg/mL. Moreover, age–stage curves further support cumulative impacts of high-concentration exposure on population process variables: a downward shift in age–stage-specific survival (*s_xj_*), reduced age-stage-specific fecundity (*f_x_*) and age-specific maternity (*l_x_m_x_*), decreased life expectancy (*e_xj_*), and a lower peak reproductive value (*v_xj_*). Notably, the peak *v_xj_* of day-8 female adults decreased from 26.55 d^−1^ in the control to 20.11 d^−1^ at 0.4 mg/mL, indicating that high SWCNT concentrations weaken the contribution of females during the reproductive prime to future population growth.

At the demographic level, *R*_0_ showed a decreasing trend with increasing concentration, suggesting cumulative negative impacts on lifetime reproductive success. More importantly, *r* and *λ* did not decline significantly at all concentrations, but only at 0.4 mg/mL (control: *r* = 0.2011 ± 0.0097, *λ* = 1.2228 ± 0.0119; 0.4 mg/mL: *r* = 0.1714 ± 0.0090, *λ* = 1.1870 ± 0.0106). Meanwhile, mean generation time (*T*) increased with concentration, consistent with developmental delay. Population projection over 60 days using TIMING-MSChart further showed a progressive suppression of growth trajectories as concentration increased; the 0.4 mg/mL group exhibited the strongest inhibition and the lowest projected number of adult females (89,094), consistent with its reduced *r* and *R*_0_. Collectively, these results indicate that the concentration threshold is not only evident at the level of individual development and reproduction but can also be amplified over longer time scales into observable differences in population growth.

These findings also provide a dose-based rationale for positioning SWCNTs in green plant protection. Because *r* and *λ* were not significantly affected at ≤0.04 mg/mL, this range could be considered a potential baseline concentration for future studies exploring SWCNT-based delivery systems (e.g., for dsRNA or other bioactive molecules). Using concentrations within this range in such applications would minimize direct confounding effects from the nanomaterial carrier itself, allowing clearer attribution of any observed biological effects to the co-delivered agent. However, direct testing of SWCNT-dsRNA complexes would be required to validate this proposition. In contrast, at higher concentrations (≥0.2 mg/mL, especially 0.4 mg/mL), SWCNTs imposed stable reproductive suppression and reduced population growth potential, implying that the material itself can exert measurable control pressure; however, this also underscores the need for stricter ecological safety boundary assessments. Notably, differences in nanomaterial type, surface chemistry, and size, as well as species-specific feeding behaviors and digestive physiology, can all contribute to heterogeneous outcomes across systems [15,31]. For example, the performance of non-functionalized SWCNTs in spider mites may differ from the reported effects of hydroxylated MWCNTs in aphid systems [32], highlighting the necessity of species- and material-specific evaluations [15].

An important consideration for translating our laboratory findings to agricultural practice is the potential discrepancy between nominal exposure concentrations and the effective doses that mites encounter under field conditions. Processes such as rainfall-induced washing, UV-mediated degradation, and interactions with leaf surface chemistry or phyllosphere microorganisms could substantially alter the persistence, bioavailability, and aggregation state of SWCNTs [3,33]. For instance, partial aggregation of SWCNTs on leaf surfaces—which may be influenced by humidity and leaf exudates—could reduce the effective concentration available for ingestion by mites, potentially attenuating the biological effects observed in our well-controlled laboratory system. Conversely, repeated applications or accumulation over time might lead to higher localized concentrations than those tested here. Therefore, while our study defines a statistically derived “no-effect” concentration threshold under controlled conditions, the practical applicability of this threshold would require validation under realistic environmental scenarios that account for these dynamic processes.

Several limitations should be acknowledged. Laboratory conditions cannot fully capture field-relevant factors (e.g., irradiation, rainfall, and foliar chemical background) that may influence SWCNT behavior and exposure [3,33,34]. In addition, potential risks to non-target organisms (e.g., natural enemies and pollinators) were not systematically evaluated [16,35]. Future work should further elucidate the molecular mechanisms underlying SWCNT effects on spider mites and, based on the defined safe window, validate the practical efficacy of SWCNT-based delivery systems under greenhouse or semi-field conditions. Meanwhile, environmental fate and non-target risk assessments should be strengthened to enhance extrapolative reliability and application relevance.

Overall, by integrating life table analysis with population projection, this study quantitatively demonstrates a concentration-dependent suppression of population growth in *T. turkestani* following SWCNT exposure and delineates a working concentration window (≤0.04 mg/mL) in which core growth parameters are not significantly affected. Fecundity became significantly suppressed at 0.2 mg/mL, while significant reductions in *r* and *λ*—and the strongest inhibition in population projection—were observed only at 0.4 mg/mL. These findings provide important guidance for dose selection in future studies exploring the use of SWCNTs in pest management strategies and contribute to defining concentration boundaries for their potential application.

## Data Availability

The original contributions presented in this study are included in the article/Supplementary Material. Further inquiries can be directed to the corresponding authors.

## Supplementary Materials

The following supporting information can be downloaded at: https://www.mdpi.com/article/10.3390/insects17030284/s1.
