# Supplementary material for: Effects of Single-Walled Carbon Nanotubes on the Development and Reproductive Performance of Tetranychus turkestani
Source: Insects. 2026 Mar 5;17(3):284. doi: 10.3390/insects17030284 (PMC13026760; doi:10.3390/insects17030284)
Supplement: Supplementary file 1 [file insects-17-00284-s001.zip › insects-4184755-supplementary.pdf]

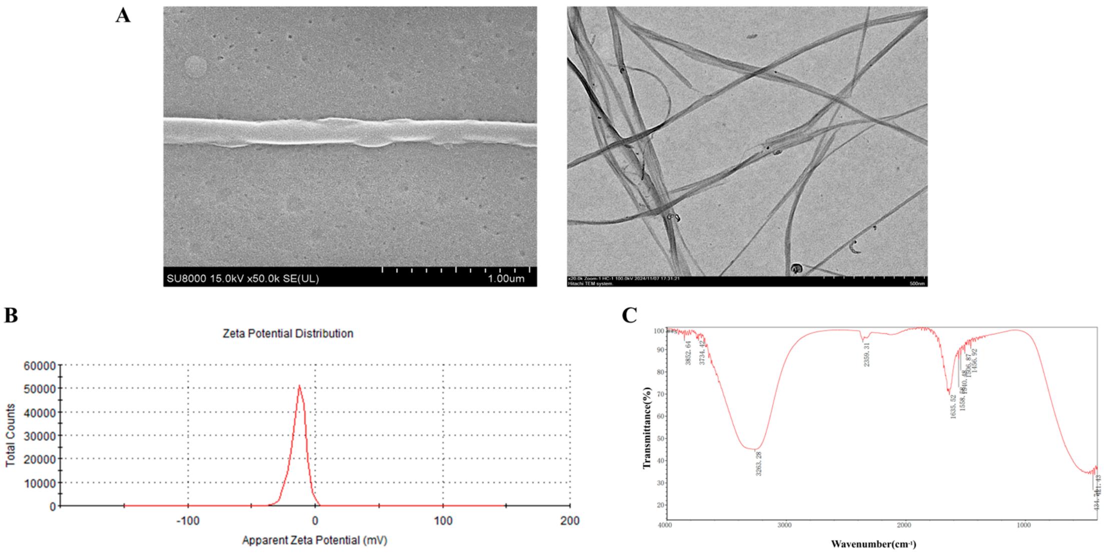

Figure S1. Characterization of the single-walled carbon nanotubes (SWCNTs). (**A**) Representative scanning electron microscopy (SEM, left) and transmission electron microscopy (TEM, right) images showing the morphology of SWCNTs. The scale bars in the SEM and TEM images represent 1 μm and 500 nm, respectively. (**B**) Zeta potential distribution of SWCNTs in aqueous suspension. (**C**) Fourier transform infrared (FTIR) spectrum of SWCNTs.
